# The associations between coronary artery disease, and non-alcoholic fatty liver disease by computed tomography

**DOI:** 10.1186/s43044-021-00222-0

**Published:** 2021-10-30

**Authors:** Samira Saraya, Mahmoud Saraya, Mohamed Mahmoud, Mohamed Galal, Hazem Hamed Soliman, Mariam Raafat

**Affiliations:** 1 Cardiovascular Department, Faculty of Medicine, Cairo University, 12 Al Saraya Street, El Manial, Cairo, P.O 11555 Egypt; 2 Radiology Department, Faculty of Medicine, Cairo University, 12 Al Saraya Street, El Manial, Cairo, P.O 11555 Egypt; 3 Students’ Hospital, Cairo University, 15 Gamal Eldin Afify street, Giza square, Giza, P.O 12511 Egypt; 4 Radiology Department, Students’ Hospital, Cairo University, 15 Gamal Eldin Afify street, Giza square, Giza, P.O 12511 Egypt

**Keywords:** Computed tomography, Coronary artery disease, Non-alcoholic fatty liver disease

## Abstract

**Background:**

Non-alcoholic fatty liver disease (NAFLD) is increasing in recognition as a hepatic condition that is unrelated to significant alcoholic consumption, but has rather, been suggested to constitute cardiovascular risk (irrespective of traditional risk factors and high-risk plaque features). Both coronary artery disease and NAFLD share the same pathophysiology and metabolic profile. NAFLD can theoretically be a source/initiator for coronary artery disease (CAD). We aimed to study the association between NAFLD, CAD, the presence of high-risk plaque features, and the severity of stenosis.

**Results:**

We recruited 800 patients with suspected obstructive CAD and planned for coronary computed tomography angiography (CCTA), Exclusion criteria: heavy alcohol consumption; contraindications to contrast media; unevaluated coronary-artery segments; other known liver disease; and use of oral corticosteroids and/or amiodarone. Non-enhanced Computed Tomography abdomen was performed before the CCTA to detect NAFLD. To study the association between NAFLD and the presence of CAD, patients were classified as to either have, or not have CAD. The CAD group were then further studied for the presence of high-risk plaque features: napkin ring sign, Positive remodelling, Low Hounsfield unit (HU), and Spotty calcium; and their association with NAFLD. Thirty-two per cent of patients had NAFLD and 45% had CAD. A significant association between NAFLD and CAD was found (OR 4.21, 95% CI (confidence interval) (2.83–6.25), *p* = 0.000). In CAD patients, significant associations were present between NAFLD and high-risk plaque features: Napkin ring sign, Positive remodelling, Low HU, and Spotty calcium (OR 7.88, 95% CI (4.39–14.12), *p* < 0.001, OR 5.84, 95% (3.85–8.85), *p* < 0.001, OR 7.25, 95% CI (3.31–15.90), *p* < 0.001 and OR 6.66, 95% CI (3.75–11.82), *p* < 0.001), respectively. NAFLD was present in 39.30%, 50.00%, 20.00%, 54.50% and 100.00% of patients with CAD; and 1–24%; 25–49%; 50–69%; 7 = 0–99%, LMD (Left Main Disease) > 50% stenosis or 3V disease, and Total occlusion, respectively, *p* < 0.001.

**Conclusions:**

NAFLD is strongly associated with CAD, high-risk plaque features and higher grade of stenosis.

## Background

Non-Non-Alcoholic Fatty Liver Disease (NAFLD) is an increasingly recognized hepatic condition that encompasses a wide spectrum of liver affection ranging from simple steatosis to advanced fibrosis, cirrhosis, and hepatocellular carcinoma. It is not essentially related to significant alcohol intake [1, 2].

Variation in the prevalence of NAFLD is found based on factors such as race, ethnicity, age, gender, geographical distribution, and the diagnostic modality used [3–5]. NAFLD has been firmly linked to insulin resistance, type 2 diabetes mellitus, dyslipidaemia, and metabolic syndrome, especially in patients with sedentary lifestyles, changing dietary patterns and increased obesity [2, 6]. These factors also comprise the risk profile for coronary artery disease (CAD). Both CAD and NAFLD share the same underlying pathophysiological mechanism, risk factors, lifestyle modification and treatment plans, and were therefore hypothesized to be closely related [7].

NAFLD is recently suggested to be an independent risk for cardiovascular diseases and is also associated with higher cardiovascular mortality [8].

Recent advances in the coronary CT (computed tomography) made it possible to diagnose obstructive CAD in acute and chronic conditions, with identification of high-risk plaque (positive remodelling, low CT attenuation, spotty calcium, or napkin ring sign). The presence of high-risk plaque was associated with a significantly increased risk of major adverse cardiac events [9]. Furthermore, non-enhanced abdominal CT has been found to have a quantitative assessment for steatosis with good sensitivity and specificity [10].

Based on these findings, we aimed to study the association between NAFLD, the obstructive CAD, the presence of high-risk coronary plaque as depicted by CT, and the severity of stenosis in an Egyptian cohort.

## Methods

Eight hundred patients with clinically suspected CAD referred to the radiology department for Coronary Computed Tomography Angiography (CCTA) were enrolled. Demographic data and medical history with special emphasis on cardiovascular risk factors (systemic arterial hypertension, diabetes mellitus, dyslipidaemia, and smoking status) were collected from all subjects. The study protocol conformed to the ethical guidelines of the 1975 Declaration of Helsinki and was approved by the Institutional Review Board of Radiology. A written informed consent was obtained from all participants before enrolling.

### Exclusion criteria

Patients presented with any of the following factors were excluded: (1) history of consumption of more than 20 g of alcohol per day; (2) contraindications to contrast media “e.g. history of reactions to any of the contrast agents, pregnancy, treatment of thyroid disease with radioactive iodine, and chronic or acutely worsening renal disease”; (3) unevaluated coronary-artery segments owing to motion artefacts or inadequate contrast medium filling; (4) other known liver diseases (carriers of the hepatitis B virus or hepatitis C virus); (5) use of oral corticosteroids and/or amiodarone that may be implicated in causing fatty liver at test time.

### CT assessment of NAFLD

Two senior radiologists with over ten years of experience in abdominal CT (M.R. and M.M.) were blinded to the results of CCTA and performed measurements of hepatic and splenic CT attenuation on the non-enhanced CT scans independently.

The hepatic CT attenuation was measured by obtaining the mean of the attenuation values for three circular regions of interest with an area of at least 2 cm^2^ that were placed at three different hepatic levels. Areas of hepatic vascular and biliary structures were avoided. The splenic CT attenuation was calculated similarly.

NAFLD was defined as either hepatic CT attenuation minus splenic CT attenuation of less than 1 HU (Hounsfield unit), or the mean CT number ratio of liver-to-spleen parenchyma of less than 1 HU [11, 12].

### CCTA analysis and high-risk coronary plaque assessment

Conventional CCTA was performed using a 64-detector row scanner (Toshiba, Aquilion TSX-101A). The images were then analysed and evaluated by two experienced senior radiologists (S.S and H.H.). The protocol followed the Society of Cardiovascular CT guidelines, which started by calculation of calcium score employing non-enhanced CT followed by contrast enhanced CCTA [13]. Coronary segments were then evaluated for the presence of coronary atherosclerotic plaque and the presence stenosis. The severity of the stenosis was categorized based on Coronary Artery Disease Reporting and Data System (CAD-RADS) classification [14].

The presence of any high-risk plaque feature such as positive remodelling, spotty calcium, napkin ring sign or low CT number was reported blindly to the presence/absence of NAFLD [15].

### Patients categorization and classification

Patients were classified into two groups; the first group were those who were diagnosed with CAD (CAD-RADS 1 or more), and the second group were those who had no CAD (CAD-RADS 0) as shown by CCTA.

Subgroup analysis of the first group regarding the severity of stenosis and the presence of high-risk plaque features was then performed.

## Results

### Statistical methods

Microsoft excel 2013 was used for data entry, and the SPSS (statistical package for social science) version 24 was used for data analysis. All collected inquiries were revised for competences and logical consistency.

Simple descriptive statistics (arithmetic mean and standard deviation) was used for the summary of normal quantitative data and frequencies used for qualitative data. The bivariate relationship was displayed in cross-tabulations, and comparison of proportions was performed using the Chi-square and Fisher’s exact tests, where appropriate. Independent T-test, one-way ANOVA (ANalysis of Variance) and post hoc tests were used to compare normally distributed quantitative data. The level of significance was set at probability (P) value < 0.05. The factors that were significantly associated with CAD and NAFLD in bivariate analysis (*p* < 0.05) were included in multivariate logistic regression models.

### Patients’ characteristics

**Fig. 1:**
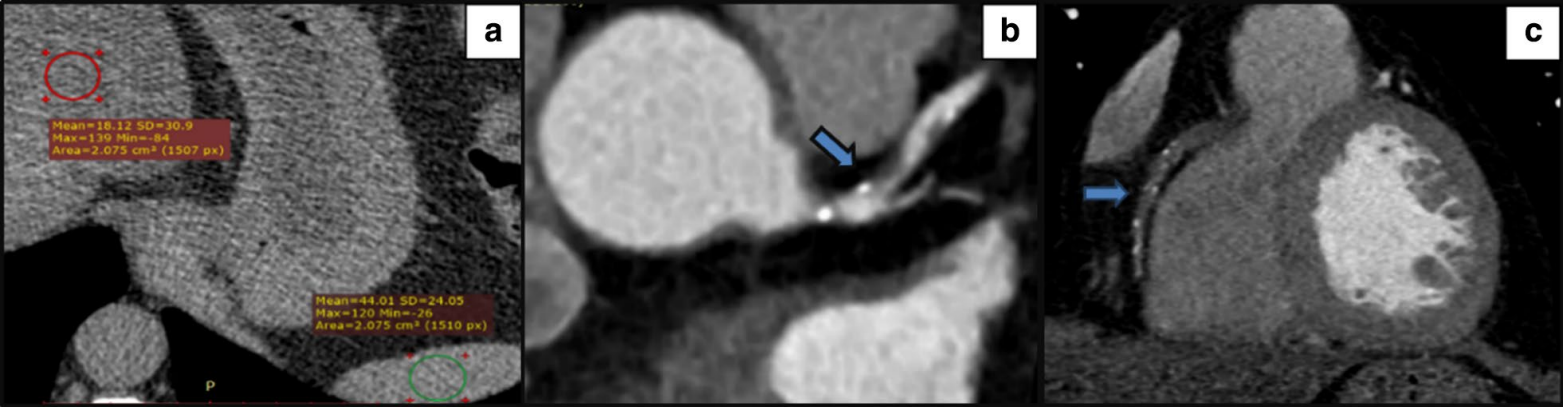
Hypertensive male patient 55 years old. **a** Axial non-enhanced CT image demonstrates attenuation measurements of the liver and spleen showing fat accumulation in the liver, with a mean liver attenuation of 18.1 HU and a mean spleen attenuation of 44 HU. **b** Coronary CT angiography shows partially calcified plaque with a low CT number (17 HU) (arrow) and positive remodelling and **c** Coronary CT angiography shows totally occluded RCA with multiple spotty calcifications (arrow)

**Fig. 2:**
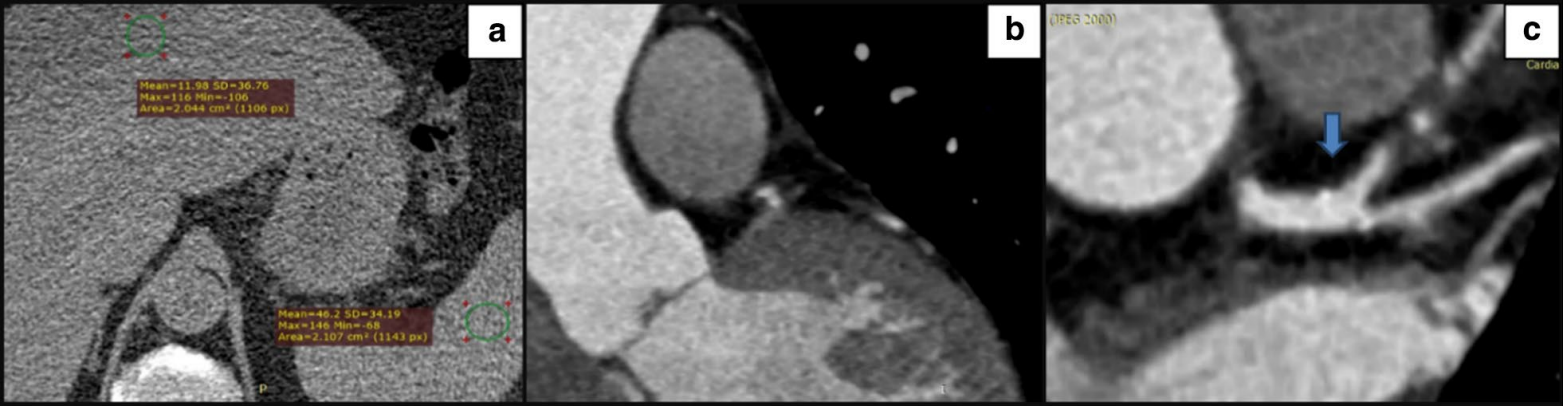
A male patient 67 years old, hypertensive, diabetic, and smoker. **a** Axial non-enhanced CT demonstrates attenuation measurements of the liver and spleen with a mean liver attenuation of 11.9 HU and a mean spleen attenuation of 46.2 HU. **b** Coronary CT angiography images shows a non-calcified plaque demonstrating a napkin-ring sign with a central low-attenuation area, surrounded by a peripheral rim of higher attenuation. **c** Coronary CT angiography images shows a calcified ostial lesion with spotty calcifications (arrow) of left main coronary artery

**Fig. 3:**
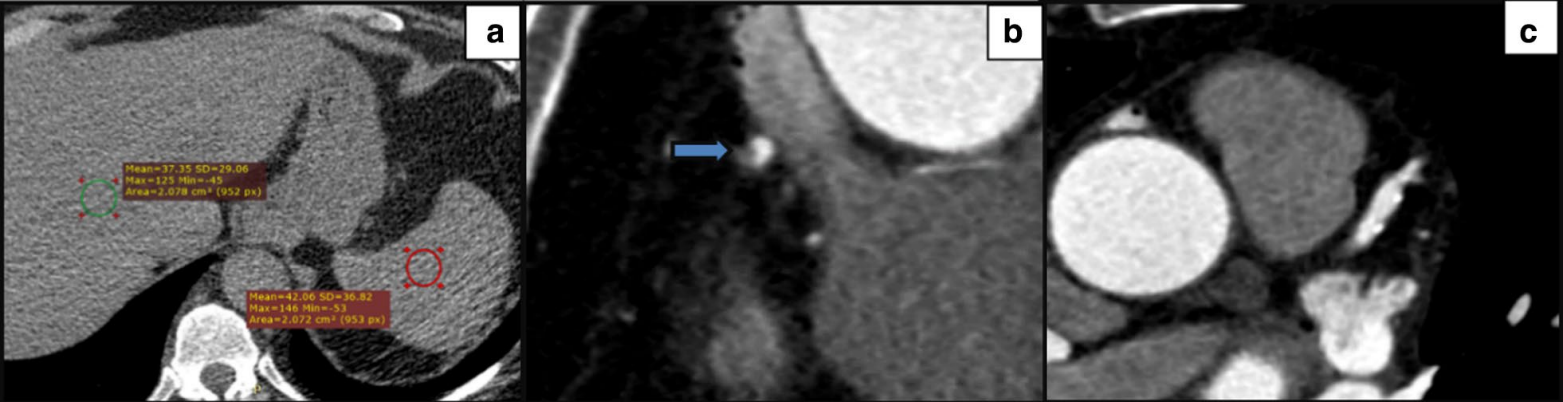
Diabetic male patient 67 years old. **a** Axial non-enhanced CT image demonstrates attenuation measurements of the liver and spleen shows a diffuse fat accumulation in the liver, with a mean liver attenuation of 37.3 HU and a mean spleen attenuation of 42.5 HU. **b** Coronary CT angiography images shows a non-calcified plaque demonstrates a napkin-ring sign (arrow) with a central low-attenuation area, surrounded by a peripheral rim of higher attenuation and **c** Coronary CT angiography images shows a plaque with spotty calcium

**Fig. 4:**
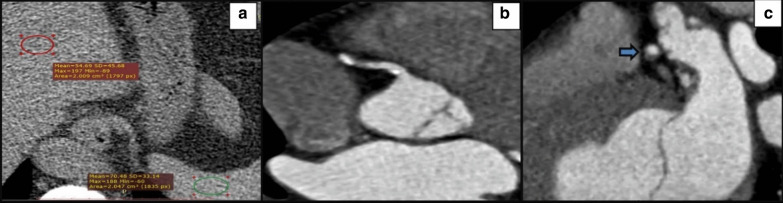
A 65-year-old lady with diabetes shows: **a** Axial non-enhanced CT image demonstrates attenuation measurements of the liver and spleen shows a diffuse fat accumulation in the liver, with a mean liver attenuation of 54.6 HU and a mean spleen attenuation of 70.4 HU. **b** Coronary CT angiography images shows a plaque with spotty calcium and **c** Coronary CT angiography shows a non-calcified plaque demonstrates a napkin-ring sign(arrow) with a central low-attenuation area, surrounded by a peripheral rim of higher attenuation

The ages of our study population ranged from 21 to 79 years with a mean of 52.46 ± 11.02 years, majority were males, and NAFLD was the most frequent clinical risk factor in the population. Ten per cent of our study subjects had calcium scores of more than 100. The frequencies of clinical risk factors for CAD are demonstrated in Table 1. Examples for patients demonstrating NAFLD and high-risk plaque features of the coronaries are depicted in Figs. 1, 2, 3, 4.

**Table 1:** Patients’ characteristics

	*n*	(%)
Male gender	568	71%
NAFLD	256	32%
Htn	232	29%
DM	168	21%
Smoking	96	12%
Dyslipidaemia	174	21.8%
Calcium score (AU)		
0	424	53%
1–100	296	37%
101–300	48	6%
> 300	32	4%

AU, Agatston units; DM, diabetes mellitus; Htn., Hypertension

### Comparison between patients with CAD and non-CAD

CAD was present in 440 (55%) subjects among the entire study group. Their demographics and risk factors of both groups are outlined in Table 2.

**Table 2:** Comparison between CAD and non-CAD patients

	CAD (*n* 440)	Non-CAD (*n* 360)	*p* value
	*n* (%) Mean (± SD)	*n* (%) Mean (± SD)	
Age (years)	55.28 ± 10.19	49.01 ± 11.031	< 0.001
Male Gender	328 (74.5%)	240 (66.7%)	0.015
NAFLD	200 (45.5%)	56 (15.6%)	< 0.001
Htn	144 (32.7%)	88 (24.4%)	0.01
DM	120 (27.3%)	48 (13.3%)	< 0.001
Smoking	64 (14.5%)	32 (8.9%)	0.014
Dyslipidaemia	126 (28.6%)	48 (13.3%)	< 0.001

CAD, coronary artery disease; DM, diabetes mellitus; Htn., Hypertension; NAFLD, non-alcoholic fatty liver disease

### Regression analysis for the presence of CAD

Direct logistic regression was performed to assess the impact of several factors on the likelihood that patients would have CAD. The model contained seven independent variables: age, sex, NAFLD, Htn. (Hypertension), DM (Diabetes Mellitus), smoking & dyslipidaemia. The full model containing all factors was statistically significant, X2 (7, 800) = 185.14, *p* value < 0.001, indicating that the model was able to distinguish between patients with and without CAD. The model explained between 20.7% (Cox and Snell R square) and 27.6% (NagelKerke R squared) of the variance in CAD status and correctly classified 70.8% of cases. As shown in Table 3, only four of the independent variables made a unique statistically significant contribution to the model (age, sex, NAFLD & smoking). The strongest independent predictor of having CAD was NAFLD, recording an odds ratio (OR) of 4.21 and controlled for all other factors in the model.

**Table 3:** Multivariate logistic regression predicting the likelihood of reporting CAD:

	OR (95% CI)	*p* value
Age (years)	1.08 (1.06–1.1)	0.000
Male Gender	1.69 (1.18–2.41)	0.004
NAFLD	4.21 (2.83–6.25)	0.000
Htn	0.68 (0.46–1.02)	0.059
DM	2.24 (0.92–5.13)	0.055
Smoking	3.07 (1.76–5.37)	0.000
Dyslipidemia	0.81 (0.36–1.85)	0.616

DM, diabetes mellitus; Htn., Hypertension; NAFLD, non-alcoholic fatty liver disease

### Subgroup analysis of CAD patients

Approximately 25% of patients had significant CAD as shown by CCTA, with more than 70% coronary artery narrowing. Table 4 Calcified and noncalcified plaques were strongly associated with NAFLD among patients with CAD (OR 12, 95% (Confidence Interval) CI 5.632–25.568, *p* value < 0.001, OR, 95% CI 2.315, 1.571–1.067, *p* value 0.022).

**Table 4:** Percent stenosis and plaque characteristics in patients with CAD

	n	%
Percent coronary stenosis		
1–24% stenosis	224	50.9%
25–49% stenosis	64	14.5%
50–69% stenosis	40	9.1%
70–99% stenosis—1or 2 vessel disease	64	14.5%
LMD > 50%—3VD	24	5.5%
Total occlusion	24	5.5%
Calcified plaques	352	80%
Non-calcified plaques	168	38%
High-risk features		
Napkin ring sign	88	20%
Positive remodelling	184	42%
Low HU	48	11%
Spotty calcium	336	76%

3VD, three vessel disease; CAD, Coronary artery disease; HU, Hounsfield unit; LMD, left main disease

High-risk plaque features (napkin ring sign, positive remodelling, low HU, and spotty calcium) were studied among the different clinical risk factors. NAFLD was found to be associated with an increased likelihood of having high-risk features among patients with CAD (Table 5).

**Table 5:** Adjusted Prevalence odd’s ratio of high-risk plaque features according to clinical risk factors (Male gender, NAFLD, Htn., DM, smoking and Dyslipidaemia) in 440 patients with CT findings of coronary artery disease

	Napkin ring sign (88)		Positive remodelling (184)		Low HU (48)		Spotty calcium (336)	
	OR 95% CI	*p* value	OR 95% CI	*p* value	OR 95% CI	*p* value	OR 95% CI	*p* value
Males	1.69 (0.94–3.05)	0.08	2.16 (1.36–3.43)	0.001	0.854 (0.82–0.89)	< 0.001	3.64 (2.27–5.84)	< 0.001
NAFLD	7.88 (4.39–14.12)	< 0.001	5.84 (3.85–8.85)	< 0.001	7.25 (3.31–15.9)	< 0.001	6.66 (3.75–11.82)	< 0.001
Htn	1.99 (1.23–3.21)	0.004	0.59 (0.39–0.89)	0.012	2.267 (1.24–4.15)	0.007	0.72 (0.45–1.13)	0.154
DM	1.71 (1.04–2.82)	0.032	1.31 (0.86–2.00)	0.207	1.385 (0.73–2.63)	0.318	2.47 (1.38–4.40)	0.002
Smoking	0.53 (0.24–1.15)	0.105	2.69 (1.55–4.64)	< 0.001	1.2 (0.53–2.70)	0.659	0.92 (0.50–1.69)	0.781
Dyslipidaemia	2.14 (1.32–3.49)	0.002	1.6 (1.05–2.42)	0.028	1.745 (0.94–3.24)	0.075	2.68 (1.50–4.78)	0.001

DM, diabetes mellitus; Htn., Hypertension; NAFLD, non-alcoholic fatty liver disease

Compared to patients without napkin ring sign, patients with Napkin ring sign were significantly older (57.9 ± 7.4 vs 54.63 ± 10.7 years, *p* value 0.001). This finding was not found with other CAD high-risk features including positive remodelling (54.6 ± 9.8 vs 55.8 ± 10.4 years, *p* value 0.2), low HU (55.8 ± 7.6 vs 55.2 ± 10.47 years, *p* value 0.608) and spotty calcium (55.4 ± 9.8 vs 55.1 ± 11.4 years, *p* value 0.793), There was no association found between the presence of NAFLD and a high calcium score of over 100 AU (Agatston Units) (OR 0.76, 95% CI 0.46–1.25, *p* value 0.279).

**Fig. 5:**
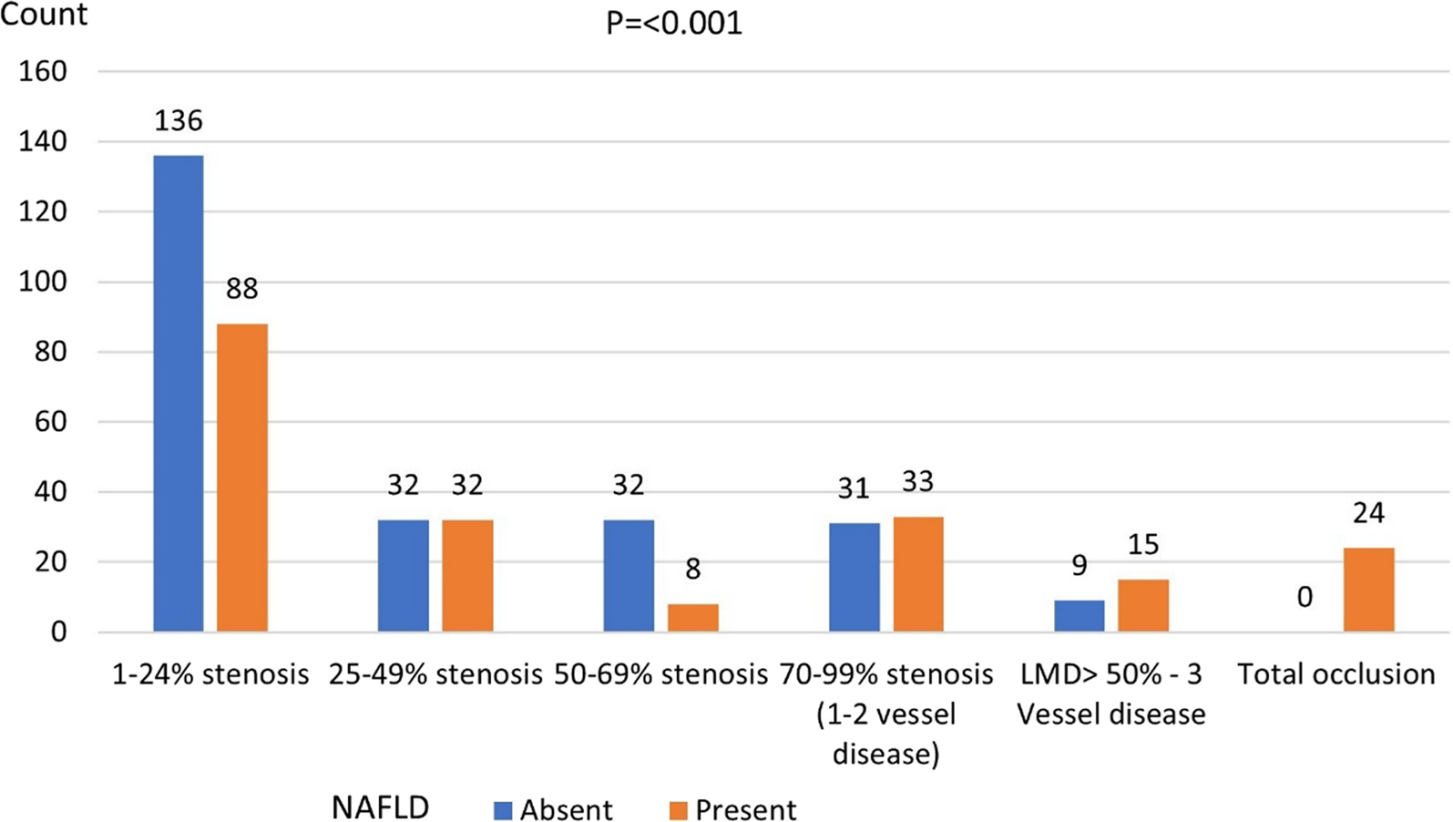
Comparison between patients with NAFLD and without NAFLD based on the severity of coronary stenosis, LMD; Left main disease. NAFLD; Non-Alcoholic fatty liver disease

There was a significant difference among groups with different severity of stenosis, and NAFLD was present in 39.30%, 50.00%, 20.00%, 54.50% and 100.00% in patients with CAD and 1–24%, 25–49%, 50–69%, (70–99% stenosis, LMD (Left Main Disease) > 50% or 3 V disease), and Total occlusion, respectively, *p* < 0.001 (Fig. 5).

## Discussion

Recent increased awareness of the NAFLD deemed essential as it has been shown to correlate with other diseases, in particular atherosclerotic CAD [16, 17]. It has been suggested that NAFLD induces a systemic inflammatory response, increased oxidative stress, insulin resistance, fatty acid toxicity and endothelial dysfunction; all of which share the same pathophysiology for development of atherosclerotic CAD [3, 18, 19].

Despite being currently considered one of the leading liver diseases with an estimated prevalence of 15%-40% in the general population [3, 20], high variations in NAFLD prevalence are observed among different populations owing to several factors such as socio-economic status, ethnicity, the method of diagnosis, and reporting of the disease [7, 21].

NAFLD is usually defined as a deposition of lipid in more than 5% of the liver in the absence of enduring or recent heavy alcohol consumption (> 20gm/day), and after exclusion of other drugs and medical conditions that can enhance this phenomenon [22].

The prevalence of NAFLD in our study population was 32%, where the study cohort showed a male gender prevalence representing 71% of patients.

Our study is considered the largest in Egypt to examine the association between NAFLD and CAD, as it involved 800 patients. We found that there is not only a statistically significant association between CAD had NAFLD, but NAFLD was also significantly associated with the presence of a high-risk plaque feature and the grade of stenosis in patients diagnosed with CAD as detected by the CCTA. We used the CT modality unenhanced and enhanced for the diagnostic purpose for NAFLD or CAD, respectively, as it confers a one-stop-shop for the diagnosis of both diseases, respectively, with adequate diagnostic accuracy [10, 23].

In the current study, based on bivariate analysis, CAD was significantly associated with older age, male gender, NAFLD, hypertension, diabetes mellitus, smoking and dyslipidemia. However, the multivariate logistic regression analysis including the abovementioned factors, showed age, smoking and NAFLD to be the only independent risk factors for CAD.

The absence of a significant association between DM, hypertension, and dyslipidemia in the multivariate model was unanticipated, owing to the well-established relation between CAD and those major modifiable risk factors [24–26]. Our findings could be attributed to us being unaware of the duration and degree of control of those risk factors in our patients.

In our study, patients with NAFLD were almost four times more likely to have CAD. Moreover, high-risk plaque features were more frequently encountered in those patients. This can be explained theoretically by the fact that NAFLD represents an interplay among all other risk factors [7]. This finding is affirmative to Puchner et al., 2015 study, which retrospectively analysed data from the Rule Out Myocardial Infarction by Cardiac Computed Tomography II (ROMICAT II) CAD arm in patients with acute coronary syndrome [15]. However, our study was prospectively conducted and included patients with suspected acute and chronic coronary syndromes.

Ismael et al., 2020 found a similar association between NAFLD and CAD where they used the coronary angiography for coronary evaluation and FibroScan for the detection of liver steatosis tested on 100 patients [27].

We have found that NAFLD was significantly more common in patients with increased CAD severity. This finding was similar to what Sun & Lü, 2011 found [28].

We could not detect any significant association between the presence of NAFLD and a calcium score over 100 AU. In contrast to Kim et al., 2012 who found a strong relationship between NAFLD and coronary artery calcification [29]. This can be attributed to the lower number of cases in our study who had a coronary calcium score above 100 AU (only 10% of the whole cohort) and those with high calcium score obscuring segmental interpretation of coronary stenosis were excluded.

## Conclusions

The high prevalence of NAFLD and its strong association with the presence of CAD confers additional independent risk for CAD. NAFLD is significantly associated with advanced high-risk coronary plaque features and a higher grade of coronary stenosis.

## Limitations of the study

The single-centre, cross-sectional design of our study is considered a limitation to corroborate a causal or temporal relationship between NAFLD and the development of CAD. Also, the use of unenhanced CT for diagnosis of NAFLD CT might not be as accurate for detecting mild steatosis [30].

## Data Availability

The data underlying this article are available in the digital library of radiology department Cairo University and cannot be shared publicly due to patients’ privacy and confidentiality as per hospital policy.

## Abbreviations

ANOVA: Analysis Of VAriance
AU: Agatston Units
CAD: Coronary Artery Disease
CAD-RADS: Coronary Artery Disease Reporting and Data System
CCTA: Coronary Computed Tomography Angiography
CI: Confidence Interval
CT: Computed Tomography
DM: Diabetes mellitus
Htn.: Hypertension
HU: Hounsfield Unit
LMD: Left Main Disease
NAFLD: Non-Alcoholic Fatty Liver Disease
OR: Odds Ratio
ROMICAT II: Rule Out Myocardial Infarction by Cardiac Computed Tomography II
SPSS: Statistical Package For Social Science
